# A simulation study on the effects of neuronal ensemble properties on decoding algorithms for intracortical brain–machine interfaces

**DOI:** 10.1186/s12938-018-0459-7

**Published:** 2018-02-27

**Authors:** Min-Ki Kim, Jeong-woo Sohn, Bongsoo Lee, Sung-Phil Kim

**Affiliations:** 1 Department of Human Factors Engineering, Ulsan National Institute of Science and Technology, Ulsan, 44919 Republic of Korea; 2 Medical Device Development Center, Daegu-Gyeongbuk Medical Innovation Foundation, Daegu, 41061 Republic of Korea; 3 School of Energy Systems Engineering, Chung-Ang University, Heukseok-dong, Dongjak-gu, Seoul, 06974 Republic of Korea

## Abstract

**Background:**

Intracortical brain–machine interfaces (BMIs) harness movement information by sensing neuronal activities using chronic microelectrode implants to restore lost functions to patients with paralysis. However, neuronal signals often vary over time, even within a day, forcing one to rebuild a BMI every time they operate it. The term “rebuild” means overall procedures for operating a BMI, such as decoder selection, decoder training, and decoder testing. It gives rise to a practical issue of what decoder should be built for a given neuronal ensemble. This study aims to address it by exploring how decoders’ performance varies with the neuronal properties. To extensively explore a range of neuronal properties, we conduct a simulation study.

**Methods:**

Focusing on movement direction, we examine several basic neuronal properties, including the signal-to-noise ratio of neurons, the proportion of well-tuned neurons, the uniformity of their preferred directions (PDs), and the non-stationarity of PDs. We investigate the performance of three popular BMI decoders: Kalman filter, optimal linear estimator, and population vector algorithm.

**Results:**

Our simulation results showed that decoding performance of all the decoders was affected more by the proportion of well-tuned neurons that their uniformity.

**Conclusions:**

Our study suggests a simulated scenario of how to choose a decoder for intracortical BMIs in various neuronal conditions.

## Background

One of the key applications of intracortical brain–machine interfaces (BMIs) is providing a neuroprosthetic technology to restore motor functions in humans with paralysis such as the amyotrophic lateral sclerosis and the brainstem stroke [1–5]. An intracortical BMI achieves this goal by detecting and translating the movement intention of users directly from cortical neuronal signals. In spite of the high cost and the possibility of tissue damages and infection, it can make good use of high signal-to-noise ratio (SNR) of intracortical signals and rich movement-related information for fine motor control [6]. A number of non-human studies have shown real-time control of an effector in 2D or 3D spaces using intracortical BMIs [7–13]. Recent intracortical BMI studies have also demonstrated real-time, multi-degree robotic arm control in humans with tetraplegia [2–5].

Intracortical BMIs translate motor cortical activity by a decoder, a set of computational algorithms that estimates motor information from the observed firing activities of neuronal ensembles. In general, BMI decoders directly estimate kinematic parameters such as position, velocity, acceleration, direction and joint angles [2, 3, 8, 12, 14]. Many decoders rely on computational models of the motor information of cortical activity such as the tuning function, which relates the primary motor cortical activity to the hand movement direction and estimates the preferred direction (PD) characterizing a specific movement direction on a single neuron. Thus, the well-tuned neurons, which imply how single neurons well fit the specific direction, provide considerable influence for the decoding algorithm. Here, the PD represents the movement direction at which a neuron maximizes its firing rate [15]. Various decoding algorithms have been proposed for intracortical BMIs, including the population vector algorithm (PVA) [8, 16], the optimal linear estimator (OLE) [1, 7, 9, 17], and the Kalman filter (KF) [18–20]. The PVA predicts kinematic states by neuronal population characterizing various directions in vector space. It allows employing population properties of neurons intuitively. The OLE is operated based on the linear model optimizing the ordinary least squares estimator. It is known that can expect better performance than the PVA through analyzing regression residuals. The KF performs prediction and update of states through the system and observation model based on the Markov chain rule, and it is known to be optimized in real-time BMI system. To understand how different decoders work in the context of BMI, some studies have attempted to compare decoders in both offline and online circumstances [14, 21, 22]. Koyama et al. [21] compared the KF and the PVA in various conditions of neuronal ensembles in the context of the open-loop and closed-loop control and showed that the KF basically decoded neural activity better than the PVA when the PDs were not uniformly distributed. Chase et al. [22] compared the open-loop and closed-loop performance of two decoders; the OLE and the PVA. It showed that the OLE performed better than the PVA under open-loop control, whereas both decoders exhibited a similar performance level under closed-loop control where subjects could compensate for a directional bias in decoders by feedback. Kim et al. [14] reported that using the KF to decode cursor velocity improved online 2D cursor control performance compared to using the OLE to decode cursor position for an intracortical BMI in humans with tetraplegia. Yet, the previous studies focused on only particular aspects of neuronal ensemble properties to investigate performance translation from offline to online decoding, without paying much attention to influences of a variety of neuronal ensemble properties such as the uniformity and proportion of well-tuned neurons on decoding performance.

In addition to the consideration of intrinsic characteristics of individual decoders, the design of an intracortical BMI should also be concerned with practical issues arising from inconsistency of chronic intracortical recordings using microelectrode arrays. Single- and multi-unit activities detected by an array often vary over time, even across recording sessions within a single day, in terms of the number of units, the SNR and other aspects of movement-related information in each unit [23]. Non-stationarity, cortical dynamics, tissue responses to electrodes and other unknown sources may contribute to these variations. At all events, it implies that one needs to rebuild a BMI corresponding to the ensemble of neuronal units detected in a particular session. This raises a question of what decoder would best fit to a given neuronal ensemble. It will be practically advantageous if one can approximately predict the performance of a chosen decoder using neuronal ensemble data obtained from a calibration phase before undertaking the entire course of building and operating BMIs.

The present study aims to address this question by exploring a relationship between decoding performance and a range of the properties of neuronal ensembles. Understanding this relationship is important for BMIs because it is often uncertain what type of decoding algorithm to choose for maximize BMI performance given a neuronal ensemble. There are many available decoding algorithms but the choice of a decoding algorithm for a given neuronal ensemble should depend on the properties of the ensemble. However, there is lack of efforts for investigating such a relationship for BMI decoding. Thus, we believe that this study may provide a useful guideline to choose an appropriate decoding algorithm depending on the neuronal states of the individual subject. In this study, we conduct a simulation study in which the firing activities of motor cortical neurons are synthesized and evaluated in the context of intracortical BMIs to extensively explore all possible variations of the selected properties [24]. Such computer simulations allow us to investigate a number of properties of neuronal ensembles in a systematic way, which is not usually tractable using the chronic recording data with implanted arrays. The present study focuses on one of the key kinematic parameters, hand movement direction, which has been widely used in BMIs [25, 26].

The basic neuronal ensemble properties studied here include the SNR of each neuron, the uniformity of PDs across the ensemble, the proportion of well-tuned neurons in the ensemble, and the distribution of the PDs of well-tuned neurons. In particular, the effect of the proportion of well-tuned neurons has not been examined before. But we assume that decoding performance may draw upon how many well-tuned neurons are detected in an ensemble and thus consider it as a key factor in this study. Here, a well-tuned neuron is defined as a neuron whose firing activity can be well explained by hand direction information. In addition, the neuronal ensemble properties are likely to change across recording sessions as well as within sessions. As such, we also investigate the effect of temporal variation of neuronal properties on decoding performance. Specifically, we examine how time-varying changes of the PDs of individual neurons affect decoding performance [27, 28].

In this study, we opt to test three most widely used decoders for intracortical BMIs: the KF, the OLE, and the PVA [19, 21, 22, 29, 30]. Although there are numerous decoding algorithms that can be used for intracortical BMIs, we focus on linear ones, as our goal is to understand relationships between decoding performance and neuronal properties rather than in-depth analysis of computational aspects of decoders. In addition, linear decoders have their own merits such that they can be readily implemented in real time and transferred to light-weight portable BMIs [17, 31].

## Simulation procedures

In order to simulate hand direction estimation via intracortical BMIs, we assumed the followings. First, we assumed that the tuning curve of simulated cortical neurons followed a unimodal bell-shaped curve [15]. In particular, we employed the cosine tuning model that is based on a sinusoidal curve because of following directional property with single neurons [15].

Second, we assumed a linear generative model with additive white Gaussian noise when we generated neuronal spikes. Here the noise was regarded any firing activity other than that encoding movement direction. Third, we probabilistically generated a neuronal spike based on the Poisson process, is defined as:

1 $$Pr(X \le j) = \frac{{\lambda^{j} \exp \left( { - \lambda } \right)}}{j!},$$

where *j* denotes the number of spikes in interval, X is the observation. The mean parameter, *λ*, of the Poisson process was determined by the firing rate estimated from the tuning curve. Fourth, we assumed that every neuron carried its own PD. Fifth, we also assumed that no cortical plasticity phenomena take place.

**Fig. 1:**
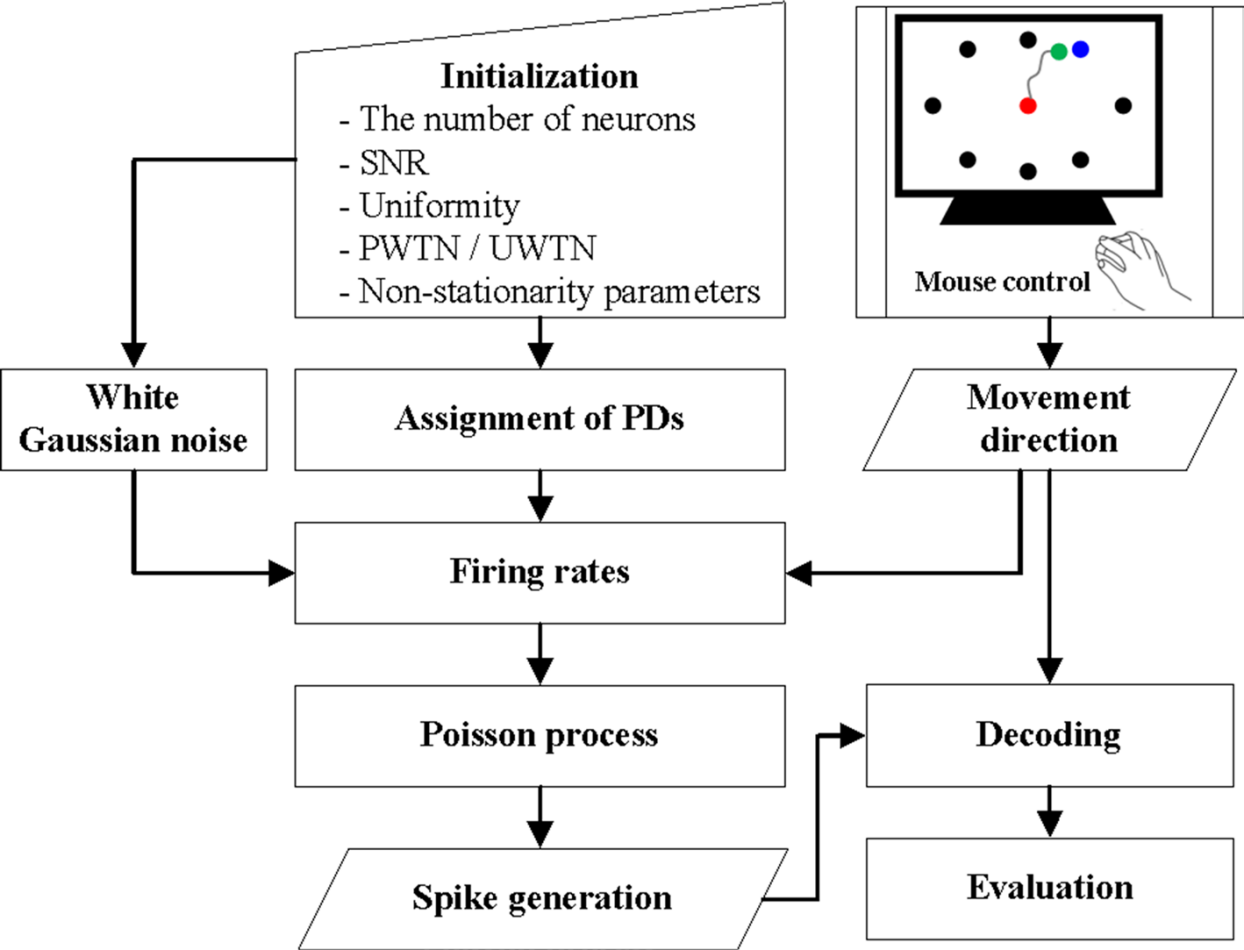
A diagram illustrating the simulation procedure. In the beginning of a simulation, we initialize the number of neurons, SNR, the uniformity of PDs, the proportion or the uniformity of well-tuned neurons, and model parameters for the non-stationarity of PDs. Then, the PD of each neuron in an ensemble is determined based on the initial conditions. The spike train of each neuron is generated using the kinematic data generated by hand movements. The spike data and hand movement data are used to build and evaluate decoding algorithms

The overall simulation procedure consisted of three steps: (1) the determination of neuronal properties, including the PD and SNR of each neuron, the uniformity of the PDs, the proportion of well-tuned neurons, the uniformity of the PDs of the well-tuned neurons and the non-stationarity of the PDs; (2) spike generation through the Poisson process; and (3) the decoding process (see Fig. 1). The details of each step are given below.

### Behavior tasks

To generate neuronal firing rates via tuning models and evaluate the performance of decoders, we created 2D hand movement data using a computer mouse (1000 dots/in., Logitech Co., USA) at a 200-Hz sampling rate. The experimenter performed the random pursuit task [1] on a preset area (30.3 cm × 30.3 cm) of a computer screen, generating angles of movement direction as diverse as possible. This task was carried out for 5 min (300 s × 20 Hz = 6000 points).

### Determination of neuronal properties

Before the start of a simulation, we determined the value of each property of a neuronal ensemble. In addition, we set the number of neurons in the ensemble. Here, we assumed that a neuron represented a single-unit or multi-unit activity recorded from the motor cortex. It has been shown that the number of neurons in practical BMIs affect decoding performance directly. In general, BMI performance increases as the number of neurons increases. The present study however did not explore the effect of the number of neurons on decoding performance, as it focused more on other neuronal ensemble properties such as the number of well-tuned neurons. For all the simulations, therefore, we fixed the number of neurons to be 60 according to the saturated performance of the previous BMI study [21].

First, we set the SNR of each neuron. Here, “signal” was defined as the firing activity of a neuron modulated by movement direction whereas “noise” as all other firing activities irrelevant to movement direction. In our simulation, firing activity was represented by a firing rate. The firing rate was used as a rate parameter for the subsequent Poisson spike generator. The firing rate of a neuron at any time instant was composed of two terms, a signal term represented by a firing rate merely modulated by movement direction, and a noise term represented by additive white Gaussian noise (AWGN). The firing rate of a neuron was calculated as:

1 $$z_{i,t} = s_{i,t} + \varepsilon_{i,t}$$

where *z*_*i,t*_ is the firing rate of a neuron *i* at time *t*, $s_{t}$ denotes the signal term and *ε*_*t*_ denotes the noise term. The SNR was defined as a ratio of the power of $s_{t}$ to that of *ε*_*t*_. Therefore, if we knew the signal power a priori then we could control the noise power (i.e. variance of AWGN) to yield a certain SNR. In our study, the SNR played a role in representing how well a neuron was tuned to movement direction.

In general, however, one cannot know this SNR before building a tuning model because *z*_*i,t*_ is only observed. One can estimate the SNR only after acquiring certain amount of neuronal spiking data together with movement data and fitting a tuning model to them. This data-driven estimate of SNR is not necessarily equivalent to the intrinsic SNR in the original firing activity of the neuron because of complex and nonlinear spike generation processes and noisy estimation of firing rates from spike trains. But since only the data-driven estimate of SNR is available to researchers in most BMI circumstances, the present study focused on the effect of the data-driven SNR, not the intrinsic SNR, on decoding performance. To this end, we attempted to have a control of the intrinsic SNR before the start of simulations to acquire a target value of the data-driven SNR. This was accomplished by creating a function that predicted the data-driven SNR from the intrinsic SNR. Instead of finding a closed-form solution to this predictive function, we empirically estimated it via a preliminary simulation in which we set a priori the intrinsic SNR (*SNR*^*Int*^), generated spikes based on it along with movement data using the cosine tuning function and the Poisson process, fitted a tuning function to the generated data and calculated the data-driven SNR (*SNR*^*DD*^) using the error variance of the tuning model. The signal power, *SP*_*i*_, of a neuron *i* was then calculated as the mean squares of the tuning function output:

2 $$SP_{i} = \frac{1}{M}\sum\limits_{t = 1}^{M} {\left( {b_{i,0} + b_{i,1} D_{x,t} + b_{i,2} D_{y,t} } \right)^{2} } ,$$

where *D*_*x,t*_ and *D*_*y,t*_ denote the x-, and y-coordinate of movement direction at time instant *t*, *b*_*i*,0_, *b*_*i*,1_ and *b*_*i*,2_ are the cosine tuning model parameters, and *M* is the number of data samples. More details of the cosine tuning model will be given below. The noise power of the neuron was estimated by:

3 $$NP_{i} = \frac{1}{\text{T}}\sum\limits_{t = 1}^{T} {e_{t}^{2} ,\quad t = 1, 2, \ldots , T } ,$$

here *NP*_*i*_ denotes a noise power on the neuron *i* at time instant *t* and *e*_*t*_ denotes estimation residual error of the tuning function. The $SNR_{i}^{DD}$ was then calculated as:

4 $$SNR_{i}^{DD} = 10\log_{10} \frac{{SP_{i} }}{{NP_{i} }} .$$

**Fig. 2:**
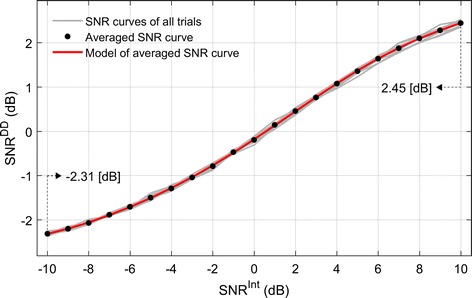
Relationship between the initial SNR (*SNR*^*Int*^) and estimated SNR (*SNR*^*DD*^). The estimated SNR is calculated by relationship between intrinsic firing activity and residual components of non-linear regression from generated spike trains

We calculated *SNR*^*DD*^ by varying *SNR*^*Int*^ in a specific range (− 10 to 10 dB) enough to represent a relationship between two variables. From the simulation results of both variables, *SNR*^*DD*^ and *SNR*^*Int*^, we empirically found that the six, or higher-order polynomials explained the relationship better than lower-order polynomials. Therefore, we chose to use the six-order polynomial to describe the relationship between *SNR*^*DD*^ and *SNR*^*Int*^ as follows:

5 $$SNR^{\text{DD}} = f\left( {SNR^{Int} } \right) = \mathop \sum \limits_{i = 0}^{6} a_{i} \left( {SNR^{Int} } \right)^{i} ,$$

where *a*_*0*_*, a*_*1*_, …, *a*_*6*_ are coefficients of the six-order polynomial model. These coefficients were empirically determined the polynomial orders from the simulated data. This outcome came from examining a mean squared error (MSE) between *SNR*^*DD*^ and *SNR*^*Int*^ with making increase the polynomial orders. Namely, we got the polynomial orders when the MSE is no decreased any more. Also, the parameters of the SNR set in our simulation were determined under practical conditions. As the variance of the *SNR*^*DD*^ was not considerable, we used the average value of *SNR*^*DD*^ obtained from the simulation. As this function provided a one-to-one mapping between *SNR*^*DD*^ and *SNR*^*Int*^, we set *SNR*^*Int*^ in the beginning to elicit a target *SNR*^*DD*^ estimated through the function. Then, we investigated how decoding performance was influenced by changes in *SNR*^*DD*^. Figure 2 shows the result of our preliminary simulation for the relationship between *SNR*^*DD*^ and *SNR*^*Int*^.

**Fig. 3:**
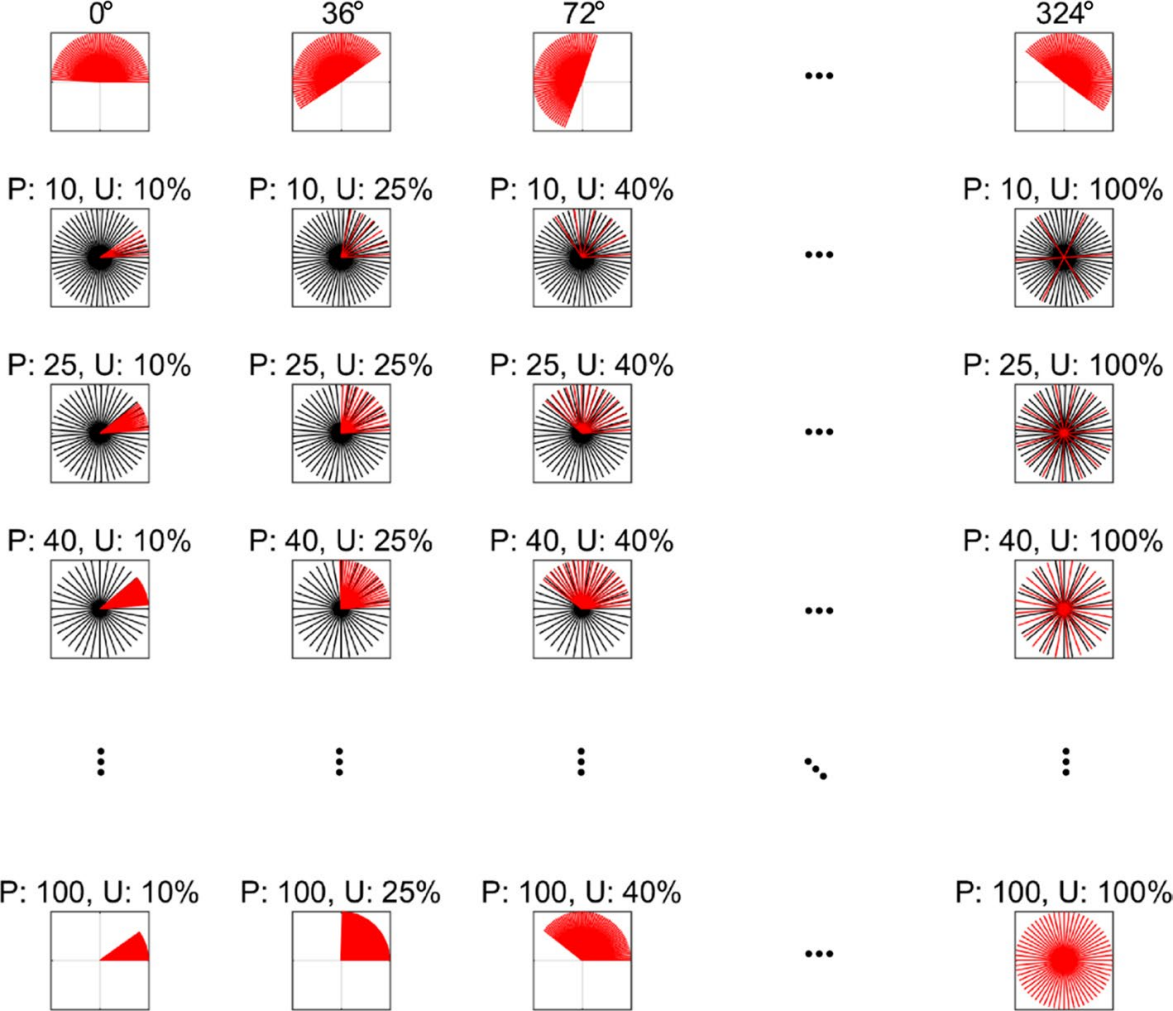
The distributions of the PDs of neuronal ensembles. Each orange or black line shows the PD of each neuron in the angular space. The orange lines denote well-tune neuron whereas the black lines denote poorly tuned neurons. The PDs of sixty neurons are displayed. (First row) Examples of the PD distributions with uniformity of 50%. Note that the PD distribution covers half of the angular space. The numbers on top indicates a bias (in degree) that is the central direction of the group of PDs. (Second—last rows) Representative examples of PD distributions of a mixture of well-tuned neurons (orange lines) and poorly tuned neurons (black lines) with various proportions of the well-tuned neurons (P) and the uniformity of these well-tuned neurons (U). Poorly tuned neurons are assumed to be uniformly distributed. P increases from the second to the last rows. U increases from the first to last columns

Second, we determined the PD of each neuron and its uniformity. The PD is defined as a 2D hand movement direction at which a neuron maximally discharges action potentials [29]. To set the PD of each neuron, we first needed to consider how to distribute the PDs among the neurons. It has been demonstrated that BMI performance could be influenced by the uniformity of the PDs across the ensemble [24]. The uniformity indicates how uniformly the PDs were distributed in the 2D angular space. Low uniformity means that neurons are tuned to similar directions covering only a part of the entire angular space. High uniformity on the other hand indicates that neurons are tuned to a wider range of directions. Here, we defined the uniformity as a percentage (%) of the whole angular space all the PDs of a neuronal ensemble occupied (see the bottom row of Fig. 3). Once the uniformity was set, PDs were set to be uniformly distributed within a given angular subspace. In this setting, we determined the central angle of uniformly distributed PDs, which was termed as a *bias* of PD (see the first row of Fig. 3). With the uniformity and the bias, we finally assigned a PD to each neuron.

Third, we determined the proportion of well-tuned neurons and their PD distribution in the ensemble. Ideally, two perfectly tuned neurons would be sufficient to decode 2D movement direction since their activities could form a basis for the 2D space (but in reality, much more than two neurons are required, if they are perfectly tuned as cosine function). Often, exploiting the activity of a small number of fairly well-tuned neurons could provide good decoding performance in BMIs. Therefore, it is important to find how many neurons are well-tuned in a given ensemble. However, it may also be equally important to know how widely the PDs of well-tuned neurons are distributed. If those PDs are distributed within a small range of the angular space, it will be still problematic to decode uncovered directions. Therefore, we encompassed this point in our simulation to investigate the effect of the proportion of well-tuned neurons (PWTN) and the uniformity of well-tuned neurons (UWTN) on decoding performance (see Fig. 3).

The well-tuned and poorly tuned neurons were determined by controlling *SNR*^*DD*^. In our simulation, the SNRs of well-tuned and poorly tuned neurons were fixed as 2.45 and − 2.31 dB, respectively. We set the PDs of poorly tuned neurons to be uniformly distributed. Figure 3 illustrates how PDs are generated depending on the uniformity and proportion of well-tuned neurons, together with uniformly distributed PDs of poorly tuned neurons.

**Fig. 4:**
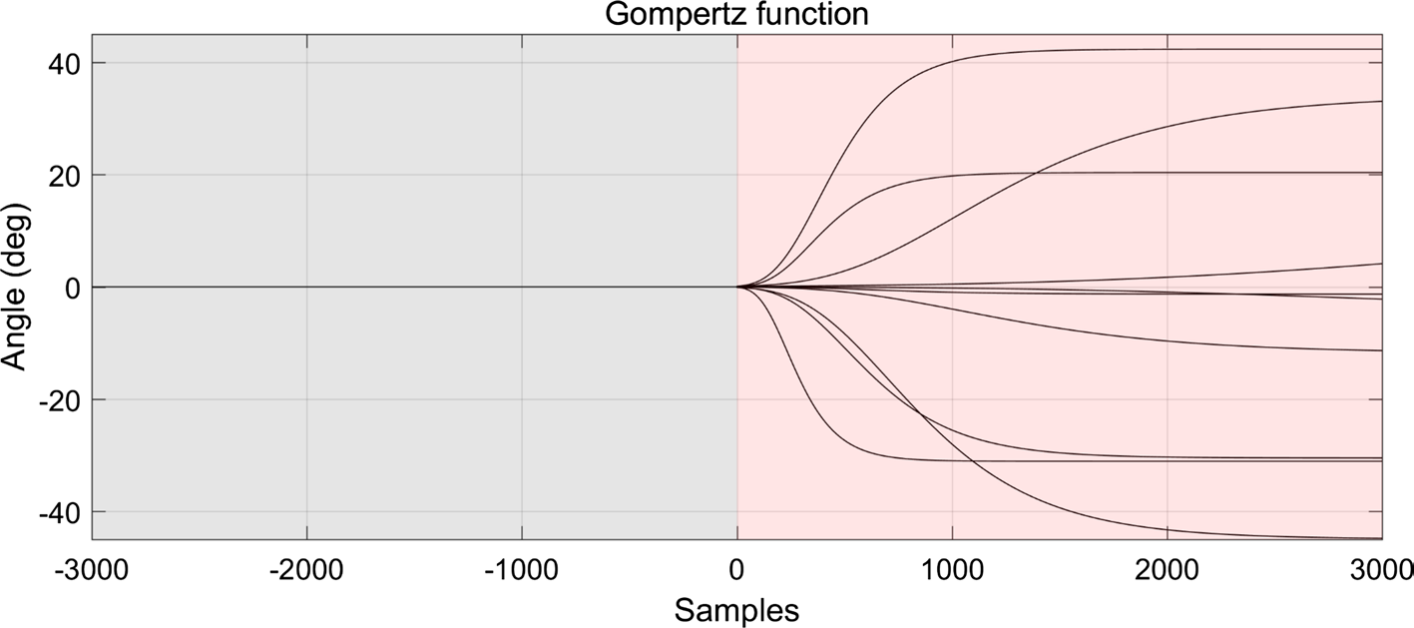
Illustrations of the non-stationarity of the PDs over time. The transparent gray color area from t = − 3000 to 0 denotes the training period in which the PDs do not change over time. On the contrary, the transparent red color area from t = 0 to 3000 denotes the test period in which the PDs gradually change over time

Fourth, we examined how non-stationary neuronal properties affect decoding performance. We implemented the non-stationarity by gradually changing PDs over time. The PD of a neuron changed according to the Gompertz model [32, 33] given by:

6 $$y_{t} = \alpha e^{{ - \lambda e^{ct} }} ,\; \quad t = 0, 1, 2, \ldots , T$$

where *y* denotes a time series of a PD and *α*, *λ*, and *c* are model parameters that determine the degree of the angular shift of a PD, the displacement along the time axis and the changing rate, respectively. The Gompertz model allows us to systematically implement the non-stationarity of the PDs by adjusting its model parameters. In our simulation, *α* was randomly chosen between − 45° and 45° and *c* was randomly chosen between 0.001 and 0.004, for each neuron. The parameter *λ* was fixed such that an angular shift began after the training period (Fig. 4). Both PWTN and UWTN were fixed to be 100%. We repeatedly evaluated the performance of the decoders for the synthetic neuronal ensemble with non-stationary PDs by randomly choosing *α* and *c* 1000 times.

### Neuronal spike generation

After the neuronal properties were determined, we generated the spikes of every neuron in a given ensemble. Given a PD of a neuron *i*, we first created a tuning coefficient vector, **b**_*i*_ = [*b*_*i*,1_ *b*_*i*,2_]^*T*^, where ||**b**_*i*_|| = 1, *b*_i,1_ = cos(PD) and *b*_i,2_ = sin(PD). Then, we used the cosine tuning function and AWGN process to synthesize the firing rate of each of *N* neurons such as:

7 $$z_{i,t} = b_{i,0} + b_{i,1} D_{x,t} + b_{i,2} D_{y,t} + \varepsilon_{t} , \quad i = 0, 1, 2, \ldots , N$$

where *z*_*i,t*_ is the firing rate of a neuron *i* at time instant *t*. *D*_*x,t*_ = cos*θ*_*t*_ and *D*_*y,t*_ = sin*θ*_*t*_ are the x- and y-coordinates of movement direction with the angle *θ*_*t*_, and *ε*_*t*_ indicates AWGN with variance *σ*^*2*^ and zero-mean. The variance *σ*^*2*^ was adjusted to produce a pre-determined *SNR*^*DD*^.

The spikes of a neuron *i* were generated by the inhomogeneous Poisson process with the firing rate *z*_*i,t*_. To generate the time series of movement direction (*D*_*x,t*_ and *D*_*y,t*_), we generated 2D hand movement data using a computer mouse control (see “Behavior tasks” section). A spike was probabilistically generated every 1 ms by the Poisson process.

### Decoding

In this study, we tested three decoders, including the PVA, the OLE, and the KF, which have been employed to decode direction from neuronal ensemble activity. The neuronal data for the decoders were the bin-count data obtained from the spike trains through the spike generation process above, with the bin width of 50 ms. These bin data as well as the 2D movement direction data were used together to train and evaluate the decoders. A total number of data points from the 5-min long hand movements was 6000. We divided the data into two halves: 50% for training and 50% for testing. A decoder was trained using the training set alone and its performance was evaluated using the test set.

The PVA decodes movement direction by linearly combining the firing activities of a population of directionally tuned neurons [16]. The PVA first estimates the PD of each neuron using the cosine tuning model. Then, it builds a population vector as a weighted sum of the PD vectors assigned to individual neurons. Here, the PD vector for a neuron is a unit vector with an angle equal to the PD of the neuron. The weight assigned to each neuronal PD vector changes every time instant and is determined by the deviation of the current firing rate from the mean firing rate of the neuron. The movement direction is then decoded as the direction of the population vector, which is given as:

8 $$\widehat{{d_{t} }} = \sum\limits_{i = 1}^{N} {\left( {z_{i} - b_{0} } \right)} c_{i} ,$$

$\widehat{d}$ denotes the population vector, *c*_*i*_ is the PD vector of neuron *i*, *z*_*i*_ indicates the current firing rate and *b*_0_ the mean firing rate.

The OLE decodes movement direction using the ordinary least squares (OLS) estimator. The optimal estimate of direction, $\widehat{d}$, is produced by the OLE as [17, 21, 22]:

9 $$\widehat{{d_{t} }} = \left( {b^{T} \varSigma^{ - 1} b} \right)^{ - 1} b^{T} \varSigma^{ - 1} z_{t} .$$

The covariance matrix, Σ, for optimizing the OLS estimator is derived from the linear regression residuals [21, 22].

The KF recursively estimates the state of movement direction using the observation and system models assuming that these models are a form of the linear Gaussian model [18, 19, 21, 30]. The KF first builds the observation model that represents the encoding of direction in neuronal ensemble, similar to the PVA:

10 $$z_{t} = H_{t} d_{t} + \varepsilon_{t}$$

A multivariate Gaussian random vector, *ε*_*t*_, represents noise with zero-mean and a covariance matrix of *Q*_*t*_. The matrix of the linear tuning model, *H*_*t*_, is estimated by the least squares method. Here we assume that *H*_*t*_ and *Q*_*t*_ are time invariant. Next, the KF builds the system model that approximates how a direction state vector changes over time with the first-order Markov process assumption:

11 $$x_{t} = A_{t} x_{t - 1} + v_{t}$$

Here, *A*_*t*_ and *v*_*t*_ are estimated again by the least square method. Once the two models are built, the KF decodes the direction state in the two steps of the prediction of the next direction state and the update of this state based on the difference between the predicted and observed neuronal activity [19, 30].

### Evaluation

To evaluate decoding performance, we compared decoded direction with the true direction of hand movements using the testing dataset. An angle difference in radians at the time index *t* of a sample (*AD*_*t*_) in the testing dataset between the decoded and true directions was calculated as:

12 $$AD_{t} = \left| {{\text{arcos}}\left( {D_{t} \cdot d_{t}^{T} } \right)} \right|$$

where *D*_*t*_ denotes the true direction of hand movements consisting of [*D*_*x,t*_ *D*_*y,t*_]^*T*^ and *d*_*t*_ is the estimated direction by a given decoder. To calculate the mean angles, we first convert *AD*_*t*_ into the rectangular (or Cartesian) coordinates of the mean angle in radian, which is calculated as:

13 $$X = \frac{1}{N}\mathop \sum \limits_{i = 1}^{N} { \cos }AD_{i} ,$$

14 $$Y = \frac{1}{N}\mathop \sum \limits_{i = 1}^{N} { \sin }AD_{i} ,$$

where *X* and *Y* denote the sum of each Cartesian coordinate from *AD*_*i*_ for *i* = 1,…, *N*. Here, *i* denotes the i-th run of decoding simulation and *N* is the number of runs (in our simulation, *N* = 100). Each run of decoding simulation was repeated 100 times by varying values of the bias that denoted the central direction of the PDs of the well-tuned neurons (see “Determination of neuronal properties” section).

The mean angle is defined as:

15 $$\theta = \tan^{ - 1} \frac{Y}{X}$$

where *θ* indicates the mean *AD*_*i*_. We tested whether *θ* was significantly different from zero using Rayleigh’s z-test (based on the probabilistic criterion via critical z-values following Zar et al.) [34]. We then compared mean ADs between decoders using Watson’s U2 test that is known as one of the methods for evaluating directional statistics [35].

Finally, we evaluated a stability of a decoder against changes in neuronal ensemble properties represented by UWTN and PWTN. The stability was defined as the variation of AD as UWTN or PWTN changed. Specifically, we calculated a difference in ADs when UWTN (or PWTN) dropped from a higher to lower levels (e.g. 100% → 80%). Then, we divided this difference by the original higher UWTN (or PWTN) level to depict the amount of changes in AD according to a decrease in UWTN (or PWTN). We repeatedly measured this by dropping UWTN (or PWTN) levels successively and averaged the measures. The resulting mean AD was defined as a variation of AD and represented a stability of a given decoder against changes in UWTN (or PWTN). Then, we performed two-way analysis of variance (ANOVA) with Bonferroni correction for multiple comparisons to compare stabilities between decoders. In other words, we analyzed the effect of decoder type and the condition of PWTN (or UWTN) on the variation of AD against changes in UWTN (or PWTN). A lower variation of AD indicated a higher stability of a given decoder.

## Results

**Fig. 5:**
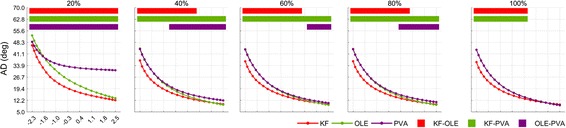
Mean change of decoders’ ADs on neuronal SNR and uniformity. These panels depict the AD change of each decoder depending on SNR change (from − 2.31 to 2.45 dB) of the neuronal ensemble with fixed uniformity (from 20 to 100%, step of the 20%). Solid red line denotes AD of the KF, green is that of the OLE, and purple is that of the PVA. Top square-dots signify SNR ranges which are a significantly different (based on Watson’s U2 test, *p* < 0.01) between decoders. The red square-dot denotes significant range between AD of the KF and that of the OLE. The green and purple also denote significant ranges regarding the KF–PVA and the OLE–PVA. On the first panel, because SNR variation has the large interval of 0.24 dB, the purple square-dots were filled although the OLE–PVA between − 1.7 and − 1.5 dB was not significant

The simulation result of the effect of SNR along with PD uniformity on decoding performance showed that AD of each decoding algorithm decreased exponentially as the SNR increased regardless of the PD uniformity (Fig. 5). Overall, the KF performed better than the other decoders for the most SNR range in all the uniformity conditions. In particular, it was superior to the others when uniformity = 20%. The OLE and PVA were slightly better than the KF when SNR > 1.85 dB on average across uniformity. Between the KF and the OLE, AD of the KF (AD_KF_) was smaller than AD of the OLE (AD_OLE_) when SNR was low (< 1.84 dB on average across uniformity) with all the uniformity values, whereas AD_OLE_ was smaller than AD_KF_ when SNR was high (> 1.88 dB on average across uniformity) and uniformity ≥ 40% (Watson’s U2 test, *p* < 0.01). Between the KF and the PVA, AD_KF_ was smaller than AD of the PVA (AD_PVA_) when SNR was low (< 1.86 dB on average across uniformity) and uniformity was greater than or equal to 20%, whereas AD_PVA_ was smaller than AD_KF_ when SNR was high (> 1.88 dB) and uniformity was 100% (Watson’s U2 test, *p* < 0.01). Between the OLE and the PVA, AD_OLE_ was smaller than AD_PVA_ when SNR was high (> −0.73 dB on average across uniformity) for the uniformity values of 20, 40 and 80% (Watson’s U2 test, *p* < 0.01), whereas AD_PVA_ was similar to AD_OLE_ for all SNRs when uniformity = 100% (Fig. 5).

**Fig. 6:**
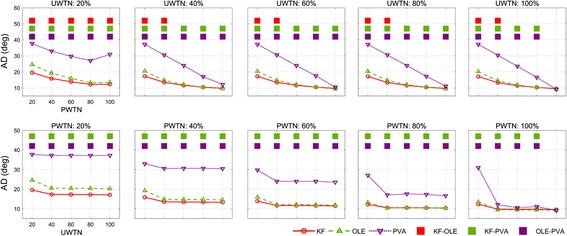
Influence of AD on the UWTN and PWTN change. Top row indicates the AD change of each decoder depending on PWTN change (from 20 to 100%) of neuronal ensemble with fixed UWTN (from 20 to 100%), whereas bottom row indicates vice versa. Solid red line denotes AD of the KF, green is that of the OLE, and the blue is that of the PVA. Top square-dots signify SNR ranges which are a significantly different (based on Watson’s U2 test, *p* < 0.01) between decoders. The red square-dot denotes significant range between AD of the KF and that of the OLE. The green and purple also denote significant ranges regarding the KF–PVA and the OLE–PVA

Next, the simulation result for the effects of PWTN and UWTN on decoding performance showed that the KF and the OLE performed significantly better than the PVA for most cases of PWTN and UWTN (Fig. 6). AD_KF_ was smaller than AD_PVA_ for all the values of PWTN and UWTN except for the cases when PWTN = 100% and UWTN ≥ 40%. (Watson’s U2 test, *p* < 0.01). AD_OLE_ was smaller than AD_PVA_ for all the values of PWTN and UWTN except for the cases when PWTN = 100% and UWTN = 60 or 100% (Watson’s U2 test, *p* < 0.01). With PWTN ≥ 80% and UWTN ≥ 40%, AD_OLE_ was smaller than AD_KF_ (Watson’s U2 test, *p* < 0.01). The performance gaps between the PVA and other decoders decreased as PWTN increased for UWTN ≥ 40%. The curves of the AD for all the decoders as a function of PWTN were not changed much by UWTN when UWTN ≥ 40%. For this range of UWTN (≥ 40%), the average (across different UWTN values) differences in ADs between a pair of decoders were: AD_PVA_ − AD_KF_ = [20.93, 17.50, 11.76, 5.48, − 0.31] (°), AD_PVA_ − AD_OLE_ = [20.07, 17.11, 12.08, 6.26, − 0.44] (°), and AD_KF_ − AD_OLE_ = [− 3.08, − 1.20, − 0.42, 0.26, 0.36](°) for the PWTN values = [20, 40, 60, 80, 100] (%), respectively.

**Fig. 7:**
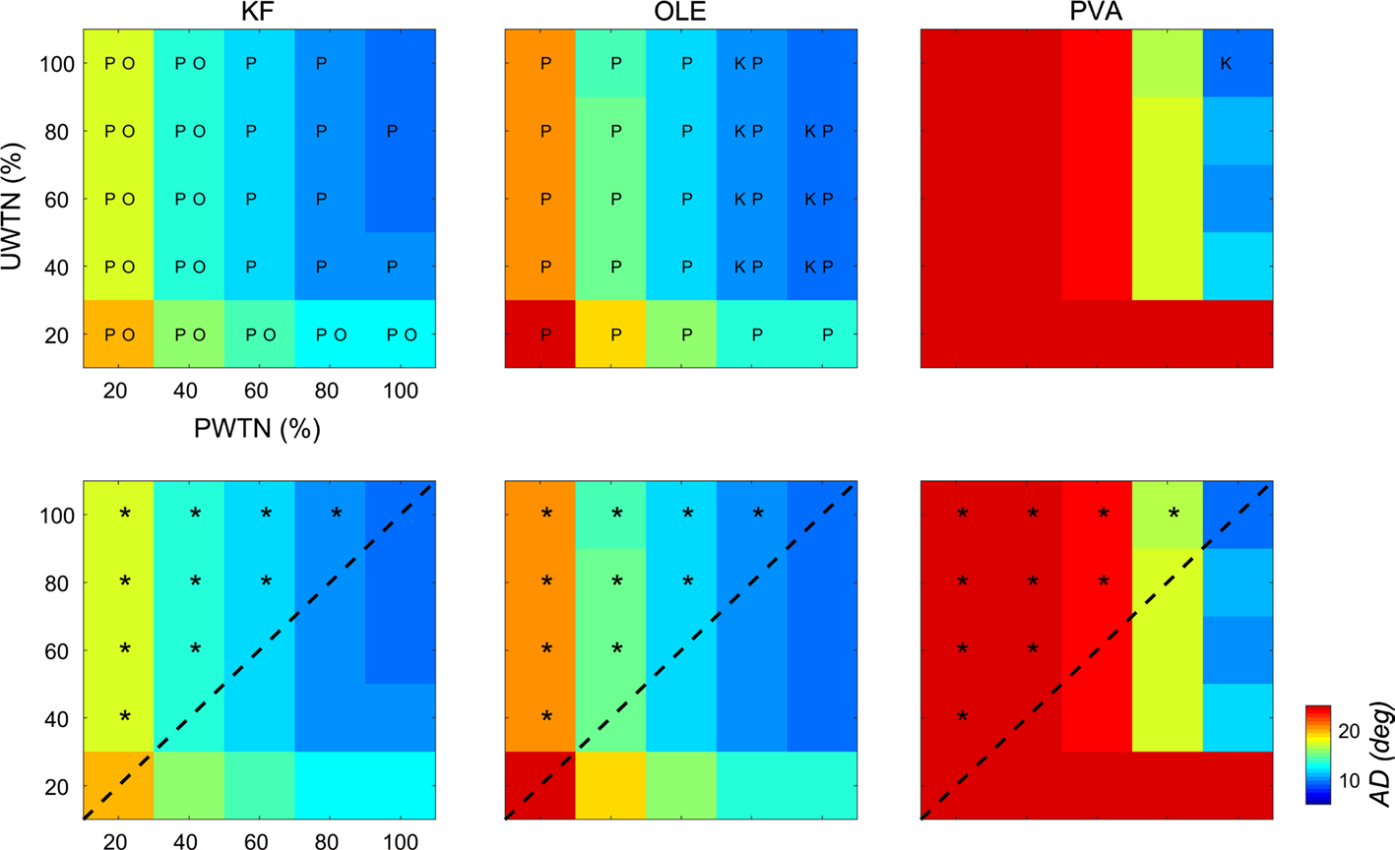
Joint space of PWTN and UWTN for each decoder. The top row contains AD topological plots, which represent the relationship between UWTN and PWTN. The character K, O, and P corresponds to the KF, OLE, and PVA, respectively, and denotes the other decoder whose performance is statistically different from the given decoder. For example, P on the KF plot indicates that the AD of the PVA is statistically different from that of the KF (*p* < 0.01). The bottom row indicates an AD difference map between an upper triangular space and a lower triangular space divided by a black-dotted diagonal boundary. Asterisks indicate that two conditions symmetric with respect to the diagonal boundary yielded statistically different ADs (Watson’s U2 test, *p* < 0.01)

We further investigated which of PWTN and UWTN influenced decoding performance more. To this end, we examined the distribution of ADs over the joint space of PWTN and UWTN for each decoder as shown in the top panel of Fig. 7. For all the decoders, an increase in PWTN seemingly improved performance more than an increase in UWTN. In other words, at any location on the 2D distribution map of ADs, moving in the direction of increasing PWTN deceased AD more than moving in the direction of increasing UWTN (Table 1). To quantify this, we performed a statistical analysis on AD differences between a pair of symmetric points with respect to the main diagonal in the 2D AD map—for example, a difference of AD between the (i, j)-th entry and the (j, i)-th entry of the map (Fig. 7, bottom). As a result, the ADs of the upper triangular points in the map, namely the points with PWTN > UWTN, were significantly smaller than those of the lower triangular points, namely the points with UWTN > PWTN, for all the decoders (Watson’s U2 test, *p* < 0.01). This implies a more crucial role of PWTN in the improvement of decoding performance compared to UWTN.

**Table 1:** Angle differences of each decoder on UWTN and PWTN change

UWTN (%)	PWTN														
	20%	40%	60%	80%	100%	20%	40%	60%	80%	100%	20%	40%	60%	80%	100%
20	19.6	15.9	13.9	12.2	12.3	24.6	19.3	16.0	13.3	13.5	37.7	33.0	29.8	27.1	31.0
40	17.3	13.6	11.7	10.5	9.8	20.5	14.9	12.1	10.4	9.5	37.2	30.6	24.0	17.1	12.2
60	17.3	13.5	11.7	10.6	9.8	20.5	14.8	12.2	10.6	9.4	37.2	30.6	24.0	17.6	10.6
80	17.2	13.4	11.6	10.5	9.8	20.4	14.7	12.1	10.5	9.4	37.2	30.6	24.0	17.3	11.2
100	17.1	13.3	11.5	10.4	9.6	20.2	14.5	11.9	10.4	9.3	37.2	30.4	23.6	16.7	9.2

**Fig. 8:**
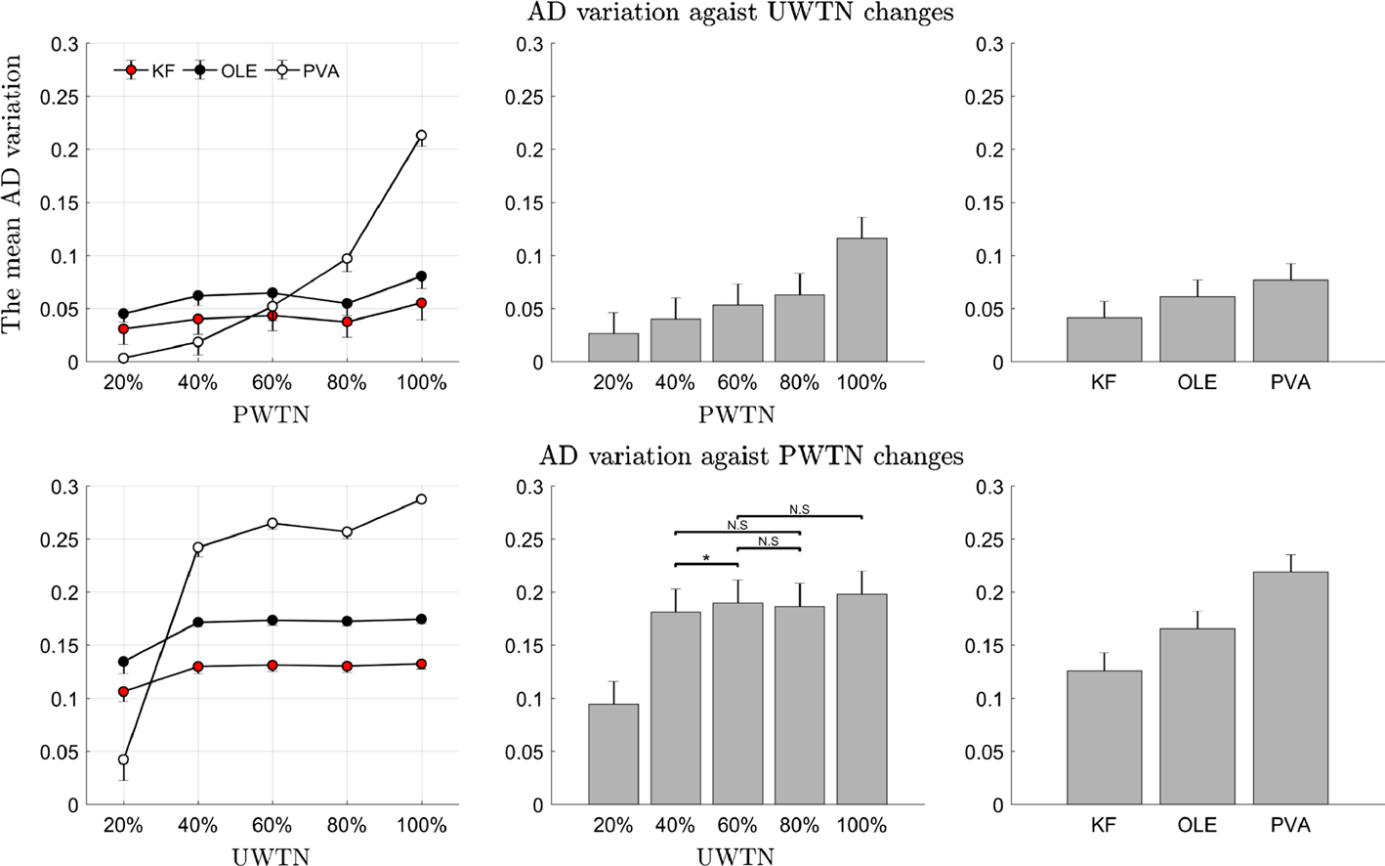
Comparison of decoding stability on UWTN or PWTN change. Top row indicates AD variation against UWTN changes and the bottom row shows that against PWTN changes. The first column depicts the AD variation of each decoder as PWTN (top) or UWTN (bottom) increased. Colored circles denote the mean AD variation of each decoder (red: KF, black: OLE, and white: PVA). The second column shows the mean AD variations for each PWTN (top) or UTWN (bottom) conditions. N.S. denotes that differences were not significant between conditions by the post hoc analysis. On the contrary, the rest unmarked bars denote significant difference (two-way ANOVA, multiple comparison tests with Bonferroni correction, *p* < 0.01). The third column shows the mean AD variations for each decoder (two-way ANOVA, multiple comparison tests with Bonferroni correction, *p* < 0.01). Error bars show the standard deviation over angle shifts (see Fig. 3)

Figure 8 depicts the stability of each decoder against changes in UWTN or PWTN. For the variation of AD against changes in UWTN, two-way ANOVA reveals the main effects of decoder type as well as PWTN on the variation of AD (*p* < 0.01). There was an interaction between decoder type and PWTN (*p* < 0.01). The KF and the OLE were more stable than the PVA when PWTN changed. For the variation of AD against changes in PWTN, two-way ANOVA reveals the main effects of decoder type as well as UWTN on the variation of AD (*p* < 0.01). It also reveals an interaction between decoder type and UWTN. The KF and the OLE were more stable than the PVA when PWTN changed from 20 to 40%. The post hoc analysis on decoder types shows that the KF was the most stable against decreases in UWTN (or PWTN), whereas the PVA was the least stable (Bonferroni correction, *p* < 0.01). In addition, the stability of the PVA against changes in UWTN was greatly affected by the condition of PWTN, which was not the case for the KF and the OLE. Another post hoc analysis on PWTN shows that the variation of AD increased as PWTN increased (*p* < 0.01). Also, the analysis on UWTN shows that the variation of AD increased UTWN changed from 20 to 40% (*p* < 0.01).

**Fig. 9:**
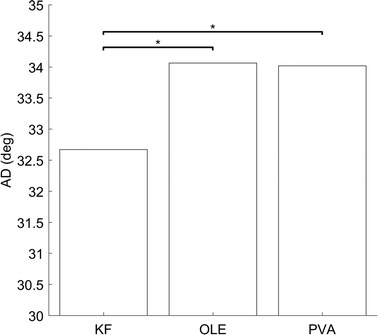
Comparison of decoders’ ADs depending on PDs change. Asterisks imply significantly different relationship (based on Watson’s U2 test, *p* < 0.01)

As stated in “Determination of neuronal properties” section, the stationary PD resulted in low ADs when it had high SNR of 2.45 dB, UWTN and PWTN of 100% [AD_KF_ = 9.62°, AD_OLE_ = 9.26°, and AD_PVA_ = 9.18°]. The AD_KF_ increased by 23.05°, whereas the AD_OLE_ and the AD_PVA_ increased by 24.8°–24.84° respectively. Consequently, the analysis on the effect of non-stationarity of PDs on decoding performance showed that the KF yielded smaller ADs than other decoders (Watson’s U2 test, *p* < 0.01), whereas there was no significant difference in AD between the OLE and the PVA (see Fig. 9). It implies that the KF was more robust to non-stationarity of PD than the other decoders.

## Conclusions and discussion

Many previous studies of the arm-reaching BMI were performed to investigate direction related neuronal tuning properties in two-or three-dimensional spaces. Mainly, directional parameters in the 2D polar coordinate are appropriate to visualize the association of neural properties, whereas those of the 3D spherical coordinate become more complex. However, 3D arm movements are more natural than 2D movements and thus represent neural tuning in a more general sense.

The main purpose of this simulation study was to investigate the influences of the various tuning properties of a neuronal ensemble on decoding performance, including the uniformity of neuronal PDs and SNR, the PWTN in an ensemble and the UTWN, and the non-stationarity of PDs. These investigations were performed by the intracortical BMI simulations, under the assumption of the recordings of the ensemble of directionally tuned motor cortical neurons. Three decoding models, including the KF, the OLE and the PVA, were tested in the simulations for estimating the hand direction.

As expected, decoding performance of all the models exponentially increased as SNR increased. With the distributions of PDs of uniformity > 20%, the KF outperformed others when SNR < − 0.48 dB whereas the OLE performed better than others when SNR > 1.42 dB. The poorer performance of the KF than others for high SNR might be due to an additional noise term of the KF [30]. Our result thus suggests that one may employ the KF with low SNRs or the OLE with high SNRs when the ensemble PDs cover more than 20% of the whole angular space. On the other hand, when the coverage of the ensemble PDs is less than 20%, the KF appears to be the best option among the three models.

As PWTN decreased, decoding performance of the PVA degraded more drastically than those of the KF and the OLE. In essence, it implies that the PVA relies more on the number of well-tuned neurons in an ensemble than other models. On the contrary, the KF and the OLE seem to exploit a small population of well-tuned neurons better than the PVA. In addition, a greater influence of PWTN on decoding performance than UTWN for all the models indicates that harvesting one more well-tuned neuron may be more crucial to direction decoding than having more widespread PDs. For instance, if one attempts to improve the performance of an intracortical BMI by enhancing directional tuning of a neuronal ensemble using a certain training paradigm, it would be better to design the training paradigm in a way of converting poorly-tuned neurons to well-tuned neurons than in a way of broadening the PDs of a fixed set of well-tuned neurons. Then, a question may arise why PWTN influences decoding performance more than UTWN. Figure 5 may provide a clue to answer for this question. It shows that AD decreases exponentially as SNR increases, implying that including well-tuned neurons with higher SNRs could be more influential to decrease ADs than increasing uniformity without increases in SNRs. We also speculate that greater impact of PWTN may be related to the algebraic characteristics of the kinematic parameter decoded here: 2-D movement direction. Theoretically, if two neurons are perfectly tuned to 2-D movement direction and work independently, they can form a basis for the 2-D space. So, modulation of their firing rates would be sufficient to reconstruct any point in the 2-D space. However, actual decoding involves estimation error of tuning model parameters due to noisy neuronal activity as well as any other unknown noise, requiring more neurons to estimate movement direction. Hence we speculate that harvesting one more well-tuned neuron would help building a more accurate basis for estimating a 2-D direction vector than simply increasing the uniformity of PDs with noisy neurons.

We also compared decoding performance of the models with respect to changes in PDs over time. The KF yielded the best performance among others, revealing its robustness to the non-stationarity of PD. Both the PVA and the OLE are dependent on linear models of each neuron whose coefficients are learned using the training data. These model coefficients are primarily determined by the PDs of neurons under the assumption of stationary data, and therefore if the PDs change after training, there is few ways the PVA or the OLE can overcome such unexpected changes. On the other hand, the KF employs the system model to predict a new state from a previous state without neuronal information, where the newly predicted state is then updated by novel neuronal data in the observation model. With this system model, the KF might have an advantage to be relatively more robust to the error from unexpected changes due to time-varying PDs.

This study demonstrates that the performance of the PVA was substantially affected by the conditions of several neuronal properties such as PWTN or SNR. Note, however, that the open-loop analysis does not always predict outcomes in closed-loop BMIs due to many other crucial factors including feedback and adaptation [21]. Thus, it is important to evaluate performance in closed-loop environments to comprehensively understand the effect of neuronal properties on decoders. However, it would still be useful to have a database to help experimenters predict the performance of a decoder before operating a BMI online, which may be made plausible by an extensive simulation study.

It is well known that decoding performance is not linearly increased as the ensemble size increases [22, 24]. Rather, performance saturates at certain point no matter how many neurons are included [36, 37]. This may indicate that now only the ensemble size itself but the properties of the neurons in the ensemble are important as a determinant of decoding performance. These facts may associate with the plasticity of cortical neurons. For example, user repetitive BMI training or experience are known to improve the decoding performance, which it may take place enhancing the neuronal plasticity and then changes the number of well-tuned neurons or their uniformity. This cortical adaptation might positively or negatively occur according to daily or temporally conditions of the subject. The present study demonstrates this by looking into the effect of the proportion of well-tuned neurons [37], which can be readily informed during a calibration stage on decoding a simple kinematic parameter (i.e. direction). Our results demonstrate that the proportion of well-tuned neurons is even more influential than the uniformity of PDs that has been generally considered as a key property for direction decoding.

The ensemble size was fixed in our simulation. However, dependency of decoding performance on various ensemble properties may be changed when the ensemble size changes. Moreover, it still remains unanswered what is more important to decoding: a few well-tuned neurons, or many mediocre neurons? If the former is correct, our focus is to select the well-tuned neurons out of all the recorded ones and extract the best information from them for decoders. If the latter is correct, we should develop a means to best exploit the information from a population of neurons. We hope that more extensive simulation studies may reveal further insights on neuronal ensemble decoding.

Although the present study explored a few basic tuning properties of a neuronal ensemble in the initialization stage of the simulation, there can be much more properties of the ensemble we can consider further. For instance, we can determine how to generate the firing rates with various directional tuning functions: e.g. von Mises function, Gaussian function as well as cosine function. Also, we can add either Poisson noise or Gaussian noise. Then, we can determine how to generate neuronal spikes with various probabilistic processes in addition to the Poisson process [38]. We can also specify correlations between neurons when generating spikes or whether the variance of the firing rate is constant or proportional to the mean. All these options can be accounted for to predict the performance a decoder and worthy of investigation. Yet, it would be also important to be concerned with the characteristics of the decoders to be analyzed and how well the synthetic data represent the realistic neuronal activities for BMIs. We anticipate that our study may provide an additional step to further investigate relationships between neuronal ensemble properties and decoding performance. Most importantly, however, it must be emphasized that the results of any BMI simulation studies should ultimately be verified in closed-loop intracortical BMIs.

## Abbreviations

BMI: brain–machine interfaces
PD: preferred direction
SNR: signal-to-noise ratio
PVA: population vector algorithm
OLE: optimal linear estimator
KF: Kalman filter
AWGN: additive white Gaussian noise
UWTN: uniformity of well-tuned neuron
PWTN: proportion of well-tuned neuron

## References

[1] Hochberg LR, Serruya MD, Friehs GM, Mukand JA, Saleh M, Caplan AH, Branner A, Chen D, Penn RD, Donoghue JP (2006). Neuronal ensemble control of prosthetic devices by a human with tetraplegia. Nature.

[2] Hochberg LR, Bacher D, Jarosiewicz B, Masse NY, Simeral JD, Vogel J, Haddadin S, Liu J, Cash SS, van der Smagt P, Donoghue JP (2012). Reach and grasp by people with tetraplegia using a neurally controlled robotic arm. Nature.

[3] Collinger JL, Wodlinger B, Downey JE, Wang W, Tyler-Kabara EC, Weber DJ, McMorland AJC, Velliste M, Boninger ML, Schwartz AB (2013). High-performance neuroprosthetic control by an individual with tetraplegia. Lancet.

[4] Aflalo T, Kellis S, Klaes C, Lee B, Shi Y, Pejsa K, Shanfield K, Hayes-Jackson S, Aisen M, Heck C, Liu C, Andersen RA (2015). Decoding motor imagery from the posterior parietal cortex of a tetraplegic human. Science.

[5] Bouton CE, Shaikhouni A, Annetta NV, Bockbrader MA, Friedenberg DA, Nielson DM, Sharma G, Sederberg PB, Glenn BC, Mysiw WJ, Morgan AG, Deogaonkar M, Rezai AR (2016). Restoring cortical control of functional movement in a human with quadriplegia. Nature.

[6] Nicolas-Alonso LF, Gomez-Gil J (2012). Brain computer interfaces, a review. Sensors.

[7] Serruya MD, Hatsopoulos NG, Paninski L, Fellows MR, Donoghue JP (2002). Instant neural control of a movement signal. Nature.

[8] Taylor DM, Tillery SIH, Schwartz AB (2002). Direct cortical control of 3D neuroprosthetic devices. Science.

[9] Carmena JM, Lebedev MA, Crist RE, O’Doherty JE, Santucci DM, Dimitrov DF, Patil PG, Henriquez CS, Nicolelis MAL (2003). Learning to control a brain–machine interface for reaching and grasping by primates. PLoS Biol.

[10] Musallam S (2004). Cognitive control signals for neural prosthetics. Science.

[11] Santhanam G, Ryu SI, Yu BM, Afshar A, Shenoy KV (2006). A high-performance brain–computer interface. Nature.

[12] Velliste M, Perel S, Spalding MC, Whitford AS, Schwartz AB (2008). Cortical control of a robotic arm for self-feeding. Nature..

[13] O’Doherty JE, Lebedev MA, Ifft PJ, Zhuang KZ, Shokur S, Bleuler H, Nicolelis MAL (2011). Active tactile exploration using a brain–machine-brain interface. Nature.

[14] Kim SP, Simeral JD, Hochberg LR, Donoghue JP, Black MJ (2008). Neural control of computer cursor velocity by decoding motor cortical spiking activity in humans with tetraplegia. J Neural Eng.

[15] Amirikian B, Georgopulos AP (2000). Directional tuning profiles of motor cortical cells. Neurosci Res.

[16] Georgopoulos AP, Schwartz AB, Kettner RE (1986). Neuronal population coding of movement direction. Science.

[17] Salinas E, Abbott LF (1994). Vector reconstruction from firing rates. J Comput Neurosci.

[18] Gilja V, Nuyujukian P, Chestek CA, Cunningham JP, Yu BM, Fan JM, Churchland MM, Kaufman MT, Kao JC, Ryu SI, Shenoy KV (2012). A high-performance neural prosthesis enabled by control algorithm design. Nat Neurosci.

[19] Wu W, Gao Y, Bienenstock E, Donoghue JP, Black MJ (2006). Bayesian population decoding of motor cortical activity using a Kalman filter. Neural Comput.

[20] Zheng X (2009). An adaptive estimation of forecast error covariance parameters for Kalman filtering data assimilation. Adv Atmos Sci.

[21] Koyama S, Chase SM, Whitford AS, Velliste M, Schwartz AB, Kass RE (2010). Comparison of brain-computer interface decoding algorithms in open-loop and closed-loop control. J Comput Neurosci.

[22] Chase SM, Schwartz AB, Kass RE (2009). Bias, optimal linear estimation, and the differences between open-loop simulation and closed-loop performance of spiking-based brain-computer interface algorithms. Neural Netw.

[23] Shain W, Spataro L, Dilgen J, Haverstick K, Retterer S, Isaacson M, Saltzman M, Turner JN (2003). Controlling cellular reactive responses around neural prosthetic devices using peripheral and local intervention strategies. IEEE Trans Neural Syst Rehabil Eng.

[24] Kim SP, Kim MK, Park GT. A simulation study on the generative neural ensemble decoding algorithms. In: Pattern recognition (ICPR). 2010 20th International Conference on. 2010. p 3797–3800.

[25] Donoghue JP, Sanes JN, Hatsopoulos NG, Gaál G (1998). Neural discharge and local field potential oscillations in primate motor cortex during voluntary movements. J Neurophysiol.

[26] Waldert S, Pistohl T, Braun C, Ball T, Aertsen A, Mehring C (2009). A review on directional information in neural signals for brain–machine interfaces. J Physiol Paris.

[27] Chestek C, Gilja V, Nuyujukian P, Foster JD, Fan J, Kaufman MT, Churchland MM, Rivera-Alvidrez Z, Cunningham JP, Ryu SI, Shenoy KV (2011). Long-term stability of neural prosthetic control signals from silicon cortical arrays in rhesus macaque motor cortex. J Neural Eng.

[28] Stevenson IH, Cherian A, London BM, Sachs NA, Lindberg E, Reimer J, Slutzky MW, Hatsopoulos NG, Miller LE, Kording KP (2011). Statistical assessment of the stability of neural movement representations. J Neurophysiol.

[29] Kalaska JF, Caminiti R, Georgopoulos AP (1983). Cortical mechanisms related to the direction of two-dimensional arm movements, relations in parietal area 5 and comparison with motor cortex. Exp Brain Res.

[30] Wu W, Black MJ, Gao Y, Bienenstock E, Serruya MD, Donoghue JP. Inferring hand motion from multi-cell recordings in motor cortex using a Kalman Filter. SAB’02-Workshop on motor control in humans and robots: on the interplay of real brains and artificial devices. 2002. p. 66–73.

[31] Kim SP, Sanchez JC, Rao YN, Erdogmus D, Carmena JM, Lebedev MA, Nicolelis MAL, Principe JC (2006). A comparison of optimal MIMO linear and nonlinear models for brain–machine interfaces. J Neural Eng.

[32] Franses PH (1994). A method to select between Gompertz and logistic trend curves. Technol Forecast Soc Chang.

[33] Franses PH (1994). Fitting a Gompertz curve. J Oper Res Soc.

[34] Zar JH (1981). Power of statistical testing: hypotheses about means. Am Lab.

[35] Mardia KV, Jupp PE. Directional statistics. Wiley series in probability and statistics. ISBN: 9780470316979. 2008. 10.1002/9780470316979.

[36] Cunningham JP, Yu BM, Gilja V, Ryu SI, Shenoy KV (2008). Toward optimal target placement for neural prosthetic devices. J Neurophysiol.

[37] Lebedev MA (2014). How to read neuron-dropping curves?. Front Syst Neurosci..

[38] Kass RE, Ventura V (2001). A spike-train probability model. Neural Comput.

